# Bypassing the systemic cardiorespiratory threshold: neuromuscular electrical stimulation elicits a SPARC response through accelerated glycolytic flux

**DOI:** 10.1007/s00421-026-06310-w

**Published:** 2026-06-29

**Authors:** Toshiaki Miyamoto, John Malone, Dominic O’Connor, Tomoyuki Katsuno, Brian Caulfield

**Affiliations:** 1 Department of Physical Therapy, School of Rehabilitation, Hyogo Medical University, 1-3-6 Minatojima, Chuo-ku, Kobe, Hyogo 650-8530 Japan; 2 Department of Health and Sport Sciences, Faculty of Science, South East Technological University, Carlow, Ireland; 3 School of Health Sciences, University of Nottingham, Queens Medical Centre, Nottingham, UK; 4 Shukugawa Katsuno Clinic, Nishinomiya, Japan; 5 Insight Research Ireland Centre for Data Analytics, University College Dublin, Dublin, Ireland; 6 School of Public Health, Physiotherapy and Sports Science, University College Dublin, Dublin, Ireland

**Keywords:** Neuromuscular electrical stimulation, Secreted protein and rich in cysteine, Myokines, Tumor suppression, Exercise, Physical activity

## Abstract

**Background:**

Secreted protein acidic and rich in cysteine (SPARC) is a myokine with potential tumor–suppressive properties. SPARC secretion typically requires vigorous-intensity exercise. Neuromuscular electrical stimulation (NMES) non-selectively recruits glycolytic Type II muscle fibers, potentially inducing intense localized metabolic stress despite a low systemic cardiorespiratory demand.

**Purpose:**

This study aimed to investigate the acute effects of a single bout of NMES at an intensity corresponding to the first ventilatory threshold (VT_1_) on circulating SPARC levels in healthy young subjects.

**Methods:**

Thirteen healthy men (21.0 ± 0.7 years) participated in a randomized crossover study: Control (rest) and 30 min NMES at the maximum tolerable intensity with a heart rate (HR) ceiling 120% of VT_1_. Serum SPARC, lactate, and glucose levels were measured before and after each session.

**Results:**

NMES significantly increased serum SPARC (616.0 ± 200.9 to 739.2 ± 279.8 ng/mL, *p* = 0.0078, *d* = 0.61) and blood lactate (1.4 ± 0.4 to 4.8 ± 1.5 mmol/L, *p* < 0.0001, *d* = 8.42) levels while reducing glucose levels (*p* = 0.0377, *d* = − 0.92). The correlation between Δ SPARC and Δ Lactate (*r* = 0.37, *p* = 0.21) showed a positive trend consistent with the effect size observed in a previous high-intensity exercise study.

**Conclusion:**

NMES at VT_1_ intensity increases circulating SPARC levels by bypassing the systemic cardiorespiratory threshold via accelerated glycolytic flux and subsequent lactate accumulation. This study provides a physiological framework for NMES as a potential strategy to induce SPARC responses in populations where strenuous volitional exertion is limited.

**Trial registration:**

UMIN-CTR (UMIN000060258).

## Introduction

Epidemiological evidence indicates that exercise and regular physical activity (PA), including light-intensity PA, significantly reduce the risk of colon cancer (Shreves et al. 2025; Amirsasan et al. 2022; Rezende et al. 2018). This protective effect is reported to be mediated via multiple physiological mechanisms, including reduced adiposity (McTiernan et al. 2019), improved insulin sensitivity, suppression of systemic inflammation (Aleksandrova et al. 2017), and regulation of immune function (Wang et al. 2025). Although these systemic changes are well-documented, the direct molecular mechanisms underlying exercise-induced tumor suppression remain largely elusive. Recent attention has focused on the muscle-as-an-endocrine-organ paradigm, where myokines secreted from contracting skeletal muscles mediate crosstalk with distant organs, including the colon (Severinsen and Pedersen 2020).

Among the diverse array of myokines, secreted protein acidic and rich in cysteine (SPARC) has been identified as one of potential candidates implicated in attenuating cancer progression (Huang et al. 2022). Mechanistic evidence from murine models suggests that SPARC inhibits the development of aberrant crypt foci, precursors to colon adenocarcinoma, by triggering apoptosis in malignant cells via the activation of the caspase-3 and − 8 pathways (Aoi et al. 2013). Notably, exercise-induced upregulation of SPARC expression appears to occur primarily in skeletal muscle, with no significant changes observed in other tissues, including the colon (Aoi et al. 2013). The concomitant increase in blood SPARC levels following exercise suggests that these circulating SPARC may originate from skeletal muscle, supporting the hypothesis that it acts as an endocrine factor that reaches the colonic environment and exerts a potential anti-tumor effect on the colon. Therefore, elucidating the dynamics of circulating SPARC in response to exercise represents a pivotal step toward understanding the physiological regulation of this tumor-suppressive myokine.

Exercise-induced circulating SPARC kinetics in humans exhibit a transient systemic release (Songsorn et al. 2017; Aoi et al. 2013), although this response appears to be strongly intensity-dependent. A previous study on healthy young men demonstrated that serum SPARC levels increased only during exercise at the second ventilatory threshold (VT_2_, 177.1 ± 10.6 bpm), where substantial lactate accumulation occurs (approximately 7.0 mmol/L), but not at the first ventilatory threshold (VT_1_, 138.9 ± 14.3 bpm), which elicits only modest lactate elevation (2.1–2.4 mmol/L) (Miyamoto et al. 2022). This intensity-dependent response was positively correlated with blood lactate concentrations (*r* = 0.411), which serves as a proxy for accelerated glycolytic metabolism. These findings suggest that reaching a specific metabolic threshold, characterized by accelerated glycolysis, is likely a prerequisite for exercise-induced SPARC secretion. Nevertheless, the requirement for VT_2_-level exertion poses a significant clinical challenge for frail populations or individuals with functional impairments, for whom such vigorous-intensity protocols are often unfeasible (Moschny et al. 2011).

Neuromuscular electrical stimulation (NMES) has gained attention as a viable alternative to conventional voluntary exercise. NMES has been extensively applied in clinical and athletic populations and has demonstrated significant improvements in skeletal muscle strength (O’Connor et al. 2020; Kern et al. 2014; Caulfield et al. 2013; Hasegawa et al. 2011), aerobic capacity (Miyamoto et al. 2016; Maddocks et al. 2016), and glucose metabolism (Miyamoto et al. 2012, 2018a). Unlike voluntary exercise, which follows the Henneman size principle (Henneman et al. 1965), the primary physiological advantage of NMES lies in its ability to recruit Type II fibers randomly or non-selectively (Maffiuletti et al. 2011; Jubeau et al. 2007). Notably, this distinct recruitment pattern occurs even at relatively low intensities, such as 20% of the maximal voluntary contraction (Gregory and Bickel 2005). Because Type II fibers rely predominantly on glycolytic metabolism, this unique recruitment pattern enables NMES to induce substantial glycogen utilization (Jubeau et al. 2007; Hamada et al. 2004) and blood lactate accumulation even at relatively low levels of systemic oxygen uptake (${{\dot{\mathrm{V}}\mathrm{O}}}_{2}$) (Miyamoto et al. 2012, 2018b). In fact, NMES has been shown to elicit a greater elevation in blood lactate levels than voluntary ergometry exercise at the same ${{\dot{\mathrm{V}}\mathrm{O}}}_{2}$ (Hamada et al. 2004). This decoupling of localized metabolic stress from systemic cardiorespiratory demand suggests that NMES may bypass the intensity thresholds typically necessitated by voluntary exercise to create the specific metabolic environment required for SPARC secretion.

However, whether the unique high local metabolic stress induced by NMES is sufficient to elevate circulating SPARC concentrations in the absence of high systemic metabolic stress remains to be elucidated. To address this gap, establishing the kinetics of SPARC at the VT_1_ threshold, an intensity where voluntary exercise fails to elicit a SPARC response due to low systemic demand (Miyamoto et al. 2022), serves as a critical proof-of-concept in a healthy adult model. This approach allows for the characterization of the pure physiological response while circumventing the metabolic heterogeneity and confounding variables, such as cachexia or pharmaceutical interventions, that are inherent in cancer populations. Therefore, this study aimed to investigate the effect of a single bout of NMES at the intensities corresponding to VT_1_ on circulating SPARC levels in healthy young subjects.

## Methods

### Participants

Healthy adult men were recruited from Hyogo University of Health Sciences. To minimize inter-individual variability in physiological responses to NMES, only male students aged 20 years or older were recruited since physiological responses to NMES are known to differ between sexes (Miyamoto et al. 2015). Eligibility required participants to be recreationally active but not enrolled in structured exercise programs, defined as < 150 min/week of aerobic exercise and < 2 days/week of resistance training (Bull et al. 2020). This target population was chosen to represent the sedentary majority, for whom increased physical activity yields the most significant health benefits. Exclusion criteria were the presence of orthopedic or neuromuscular disorders, or having implanted electronic devices (e.g., pacemakers).

### Sample size calculation

The sample size was determined based on our previous study investigating serum SPARC responses to acute exercise in a similar population (Miyamoto et al. 2022). In that study, a significant session-by-time interaction was observed for serum SPARC levels with a large effect size (partial eta squared [*η*_*p*_^*2*^] = 0.313). Using G*Power 3.1.9.7, we performed a power analysis assuming a medium-to-large effect size (Cohen’s *f* = 0.40 to be conservative), an alpha level of 0.05, and a power of 0.80. The analysis indicated that a minimum of 12 participants was required. To account for potential dropouts or technical failures during blood sampling (as seen in our previous work), we enrolled 19 participants to ensure sufficient statistical power for the linear mixed model.

### Ethical considerations

The study was conducted in accordance with the Declaration of Helsinki and approved by the Ethical Committee of Hyogo University of Health Sciences (Approval No. #15015-4). The study protocol was retrospectively registered with the UMIN Clinical Trials Registry (UMIN000060258). All participants received a comprehensive explanation of the study objectives, procedures, and potential risks before providing written informed consent.

### Experimental protocol

Participants attended the laboratory on three separate occasions, with each session separated by at least 48 h to avoid carry-over effects. During the first visit, participants performed an incremental exercise test using a cycle ergometer (Aerobike 75XL-Ⅱ, Combi, Japan) to measure their individual peak ${{\dot{\mathrm{V}}\mathrm{O}}}_{2}$ and VT_1_ for the subsequent experimental interventions. Further, participants performed two interventions: (1) Control: 30-min passive rest, lying in the supine position; (2) NMES: 30-min NMES applied to the lower body. Sessions were performed in a random order, based on simple randomization using computer-generated random lists. The participants were prohibited from performing intense exercise and consuming alcohol for 10 h before each session. Also, participants were instructed to abstain from caffeine intake on the day of the experiment to minimize its influence on autonomic and metabolic responses.

Participants arrived at the laboratory at 9:30 AM after overnight fasting prior to each session. After a 30-min rest period on a treatment bed, blood was sampled from the median cubital vein to evaluate serum SPARC concentration. The participants then performed any of the two interventions. During each session, heart rate (HR) was recorded every 5 min using an HR monitor (H10, Polar Japan, Tokyo, Japan). Immediately after each session, blood sampling for SPARC was repeated. Additionally, lactate and glucose concentrations were evaluated before and after each session. The room temperature was maintained at 24–26 °C.

### Incremental exercise test

An incremental exercise test was performed using a cycle ergometer to determine individual peak workloads and metabolic thresholds. The test protocol consisted of a 1-min rest period, 2 min of 20 W-loaded cycling as a warm-up, a progressively increasing work rate of 20 W/min until exhaustion, and a 3-min recovery phase with 20 W-loaded cycling as a cooling-down period. Throughout the test, participants were instructed to maintain a constant pedal cadence of 60 rpm, and the test was terminated when participant could no longer maintain this cadence despite verbal encouragement. The seat and handlebar heights of the cycle ergometer were adjusted for each participant prior to the test.

Respiratory gas exchange was measured using the breath-by-breath method (Aero monitor AE 300, Minato Medical Science, Tokyo, Japan) to calculate the peak ${{\dot{\mathrm{V}}\mathrm{O}}}_{2}$ and VT_1_, and electrocardiogram (ECG) telemetry was simultaneously performed using a bipolar lead system (LX5120, DS7520; Fukuda Denshi, Tokyo, Japan). Analog signals from the gas analyzer and ECG were continuously monitored and stored on a computer via an analog-to-digital converter at a sampling rate of 500 Hz. ${{\dot{\mathrm{V}}\mathrm{O}}}_{2}$, carbon dioxide production (${{\dot{\mathrm{V}}\mathrm{CO}}}_{2}$), ventilation (${{\dot{\mathrm{V}}\mathrm{E}}}$), end-tidal O_2_ and CO_2_ partial pressure (P_ET_O_2_ and P_ET_CO_2_, respectively), and HR were calculated every 10 s.

The VT_1_ was estimated using respiratory gas exchange parameters, which included the point of departure from linearity in ${{\dot{\mathrm{V}}\mathrm{E}}}$ and ${{\dot{\mathrm{V}}\mathrm{O}}}_{2}$, an abrupt increase in the expiratory O_2_ fraction, a systematic increase in ${{\dot{\mathrm{V}}\mathrm{E}}}$ / ${{\dot{\mathrm{V}}\mathrm{O}}}_{2}$ without any increase in ${{\dot{\mathrm{V}}\mathrm{E}}}$ / ${{\dot{\mathrm{V}}\mathrm{O}}}_{2}$, and a systematic increase in P_ET_O_2_ without any decrease in P_ET_CO_2_ (Peter Raven 2014; Wasserman 1984). The resting, warm-up, and cooling-down periods were excluded from the analysis.

### NMES procedure

This study used an NMES device equipped with six silicone rubber belt electrodes (Homer Ion, Tokyo, Japan), which was the same device used in our previous studies (Tamiya et al. 2024; Miyamoto et al. 2016, 2018a, b). To ensure optimal conductivity, the contact surfaces between the skin and electrodes were covered with specially designed moist cloths. The system utilized three independent channels, allowing for synchronized but separate stimulation of the waist, bilateral thighs, and bilateral lower legs. The electrodes were positioned at the waist, thighs, and lower legs as follows: (1) the waist electrode was placed above the anterior superior iliac spine; (2) the thigh electrodes were wrapped 5 cm proximal to the superior border of the patella; and (3) the lower leg electrodes were positioned 5 cm proximal to the medial malleolus. The electrical circuit was configured with the anode at the distal thigh and the cathodes at the waist and distal lower legs.

The stimulator current waveform was designed to exponentially increase the pulse with a pulse width of 250 µs. Electrical stimuli were set at a frequency of 4 Hz. In our previous study (Miyamoto et al. 2018b), NMES at a frequency of 4 Hz was shown to significantly increase blood lactate concentrations compared to a resting control session (from 1.6 ± 0.6 mmol/L to 2.9 ± 0.8 mmol/L). Since circulating SPARC responses are closely associated with lactate accumulation and accelerated glycolytic flux (Miyamoto et al. 2022), we selected this specific 4 Hz setting to maximize the potential for stimulating SPARC secretion through NMES-induced metabolic stress. This frequency has also been reported to improve metabolism and physical fitness in our previous interventional studies (Miyamoto et al. 2016, 2018a).

Because the torque production and hypertrophic effects of NMES are intensity-dependent (Natsume et al. 2018; Maffiuletti 2010), and NMES is most commonly prescribed at the maximum tolerable intensity in clinical settings, the stimulation intensity in this study was set to the highest level each participant could tolerate. Crucially, a physiological ceiling was pre-set at 120% of the HR recorded at VT_1_. This ceiling was strategically established to ensure that the systemic cardiorespiratory demand remained below the intensity typically required to stimulate SPARC secretion during voluntary exercise. In our previous study (Miyamoto et al. 2022), voluntary exercise performed at 128.4 ± 12.0% of HR at VT_1_ significantly increased serum SPARC levels, whereas exercise at 96.0 ± 11.6% of HR at VT_1_ failed to do so. By limiting the systemic response to 120% of HR at VT_1_, we specifically aimed to investigate whether NMES could bypass the systemic cardiorespiratory threshold required for SPARC release through localized high-threshold fiber recruitment.

During the initial 10-minute ramp-up period, the stimulation intensity was incrementally increased to the maximal tolerable intensity, with adjustments made every minute to account for habituation to NMES. The constant intensity for the remaining 20-minute session was determined by either the maximum tolerable stimulation intensity reached at the end of the 10-minute ramp-up or the point at which the HR reached 120% of the HR at VT_1_, whichever came first. The maximum output currents (mA) for the right upper leg, left upper leg, and bilateral lower legs were recorded.

### Blood analysis

From the median cubital vein, 4–5 mL of blood was sampled into vacuum tubes to determine the concentration of serum SPARC. After sampling, the blood was immediately centrifuged at 3,000 rpm for 10 min at 4 °C, and the collected serum was stored at -20 °C until analysis. Serum SPARC levels were measured using a solid-phase, sandwich, two-site enzyme-linked immunoassay (ELISA) with the SPARC ELISA Kit (Quantikine ELISA Human SPARC, R&D Systems, Inc., USA) and an automatic ELISA microplate reader (SpectraMax M2e, Molecular Devices Japan, Tokyo, Japan), following the manufacturer’s instructions. The intra-assay coefficient of variation for serum SPARC was 1.8–2.5%. Blood lactate and glucose concentrations were measured using an automatic analyzer (Lactate Pro Sensor and Glucocard MyDIA, Arkray, Kyoto, Japan).

### Statistical analysis

To account for the repeated-measures design and individual variability, linear mixed models using the restricted maximum likelihood method were employed. The models for each dependent variable (SPARC, lactate, and glucose) included session (Control vs. NMES) and time (Pre vs. Post) as fixed effects, as well as their interaction (session × time). Participant ID was included as a random effect. Model goodness-of-fit and assumptions were verified by inspecting residual plots and normal quantile plots to ensure homoscedasticity and normality of residuals. The Akaike Information Criterion (Portet 2020) was used to guide model selection. When a significant interaction or main effect was identified, post-hoc comparisons were conducted using Tukey’s Honestly Significant Difference (HSD) test. For all analyses, mean differences (MD) and 95% confidence intervals (CI) were reported. Effect sizes were quantified using *η*_*p*_^*2*^ for the fixed effects and Cohen’s *d* for pairwise comparisons. The magnitude of Cohen’s *d* was interpreted as small (0.2), medium (0.5), or large (0.8) (Cohen 1988).

Furthermore, to ensure internal validity, a per-protocol analysis was performed. We predefined a minimum compliance threshold of 80% of HR at VT_1_, representing the lower bound of the intensity range previously shown to be non-responsive for SPARC secretion (Miyamoto et al. 2022). Participants who could not tolerate a stimulation intensity sufficient to reach this threshold (sub-threshold group) were excluded from the final analysis to avoid confounding the results with sub-threshold metabolic stimuli. To compare baseline characteristics and NMES-related physiological parameters between the completers and the sub-threshold group, the Mann-Whitney U test was employed. This non-parametric approach was selected due to the small and unequal sample sizes, which precluded the assumption of normality.

To explore the physiological mechanisms underlying the SPARC response, correlation analyses were performed. The absolute changes (Δ) in serum SPARC, blood lactate, and blood glucose concentrations were calculated by subtracting the pre-intervention values from the post-intervention values recorded during the NMES session. The relationships between Δ SPARC and Δ Lactate, as well as between Δ SPARC and Δ Glucose, were evaluated using Pearson’s correlation coefficients (*r*). Data are expressed as mean ± standard deviation, and statistical significance was set at *p* < 0.05. All statistical analyses were performed using JMP Student Edition 19 (SAS Institute, Cary, NC, USA).

## Results

### Participant characteristics and exercise capacity

A total of 19 volunteers were initially enrolled in the study. None of the participants had previous experience with NMES. Of the 19 participants, two withdrew after the incremental exercise test due to scheduling difficulties, and one was excluded due to technical failure during blood sampling. Among the remaining 16 participants, three (sub-threshold group) were excluded from the final analysis for failing to meet the physiological compliance threshold (80% of HR at VT_1_). As shown in Table 1, there was no significant difference in body fat between the completers (15.8 ± 6.1%) and sub-threshold group (14.0 ± 3.1%). The mean peak ${{\dot{\mathrm{V}}\mathrm{O}}}_{2}$ and VT_1_ of the completers were 35.7 ± 5.1 mL/kg/min and 21.7 ± 4.4 mL/kg/min, respectively.

**Table 1:** Characteristics of study participants and physiological responses during neuromuscular electrical stimulation

		Participants (*n* = 19)	Completers (*n* = 13)	Sub-threshold group (*n* = 3)	*p*-value
Anthropometrics	Age (years)	20.9 ± 0.7	21.0 ± 0.7	20.0 ± 0	0.0354
	Height (cm)	171.9 ± 5.4	169.8 ± 3.6	180.0 ± 2.0	0.0099
	Body mass (kg)	65.1 ± 8.7	62.1 ± 8.5	70.1 ± 5.0	0.0801
	Body mass index (kg/m^2^)	22.0 ± 2.8	21.6 ± 3.0	21.6 ± 1.5	0.9463
	Body fat (%)	16.7 ± 6.0	15.8 ± 6.1	14.0 ± 3.1	0.6865
Exercise capacity	Peak ${{\dot{\mathrm{V}}\mathrm{O}}}_{2}$ (ml/kg/min)	35.9 ± 4.7	35.7 ± 5.1	38.6 ± 4.4	0.2260
	Peak HR (bpm)	191.6 ± 7.0	192.2 ± 7.8	188.3 ± 7.2	0.5413
	VT_1_ (ml/kg/min)	21.1 ± 3.9	21.7 ± 4.4	20.7 ± 4.4	0.5905
	VT_1_ (% peak ${{\dot{\mathrm{V}}\mathrm{O}}}_{2}$)	59.2 ± 11.0	61.0 ± 12.1	54.1 ± 5.5	0.3463
	HR at VT_1_ (bpm)	138.9 ± 13.4	138.7 ± 15.4	138.7 ± 12.3	> 0.9999
NMES output	Right thigh (mA)	–	121.6 ± 19.1	127.3 ± 23.7	0.7865
	Left thigh (mA)	–	117.0 ± 27.4	126.6 ± 22.5	0.5005
	Lower legs (mA)	–	129.4 ± 22.8	136.1 ± 15.7	> 0.9999
Physiological load of NMES	HR during NMES (bpm)	–	131.0 ± 13.3	103.7 ± 15.3	0.0222
	HR during NMES (% HR at VT_1_)	–	95.4 ± 13.3	74.5 ± 4.5	0.0106

$\dot{V}O_{2}$ oxygen uptake, *HR* heart rate, *VT*_*1*_ first ventilatory threshold, *NMES* neuromuscular electrical stimulation; The sub-threshold group was defined as participants who were unable to reach the target intensity (80% of HR at VT_1_) due to pain/discomfort (*n* = 3). Participants excluded for scheduling (*n* = 2) or blood collection errors (*n* = 1) are not included in this comparison. Due to the small sample size in the sub-threshold group (*n* = 3), the Mann-Whitney U test was employed for these comparisons. Data are expressed as mean ± standard deviation

### NMES intensity and physiological demands

**Fig. 1:**
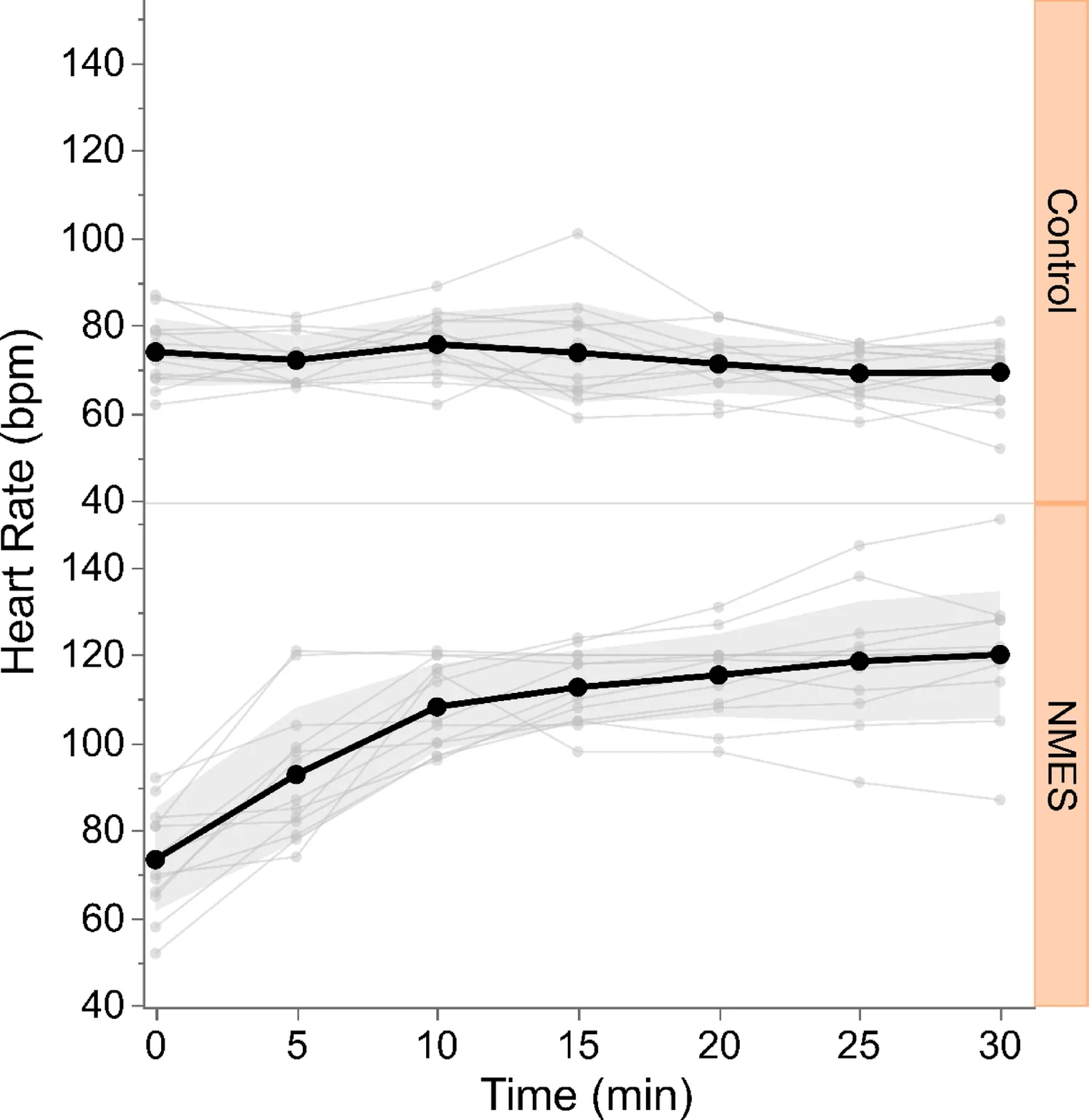
Individual and mean heart rate responses during the experimental sessions. Temporal progression of heart rate during the 30-minute Control (top panel) and neuromuscular electrical stimulation (NMES; bottom panel) sessions (*n* = 13). Thin gray lines represent individual participant data, and the thick black line with closed circles indicates the overall mean response. The shaded areas around the mean lines represent the standard deviation

The mean output pulse currents of NMES applied was 117.0–129.4 mA (Table 1). Completers’ HR during NMES was 131.0 ± 13.3 bpm (vs. Control: 70.2 ± 8.9 bpm; *p* < 0.001), which reached 95.4 ± 13.3% of the HR at VT_1_. Individual and mean temporal changes in HR during the 30-minute sessions are presented in Fig. 1. Following the initial 10-minute intensity adjustment period, although individual HR traces exhibited temporary fluctuations inherent to raw physiological responses, the overall trend generally stabilized.

### Serum SPARC and metabolic responses

**Fig. 2:**
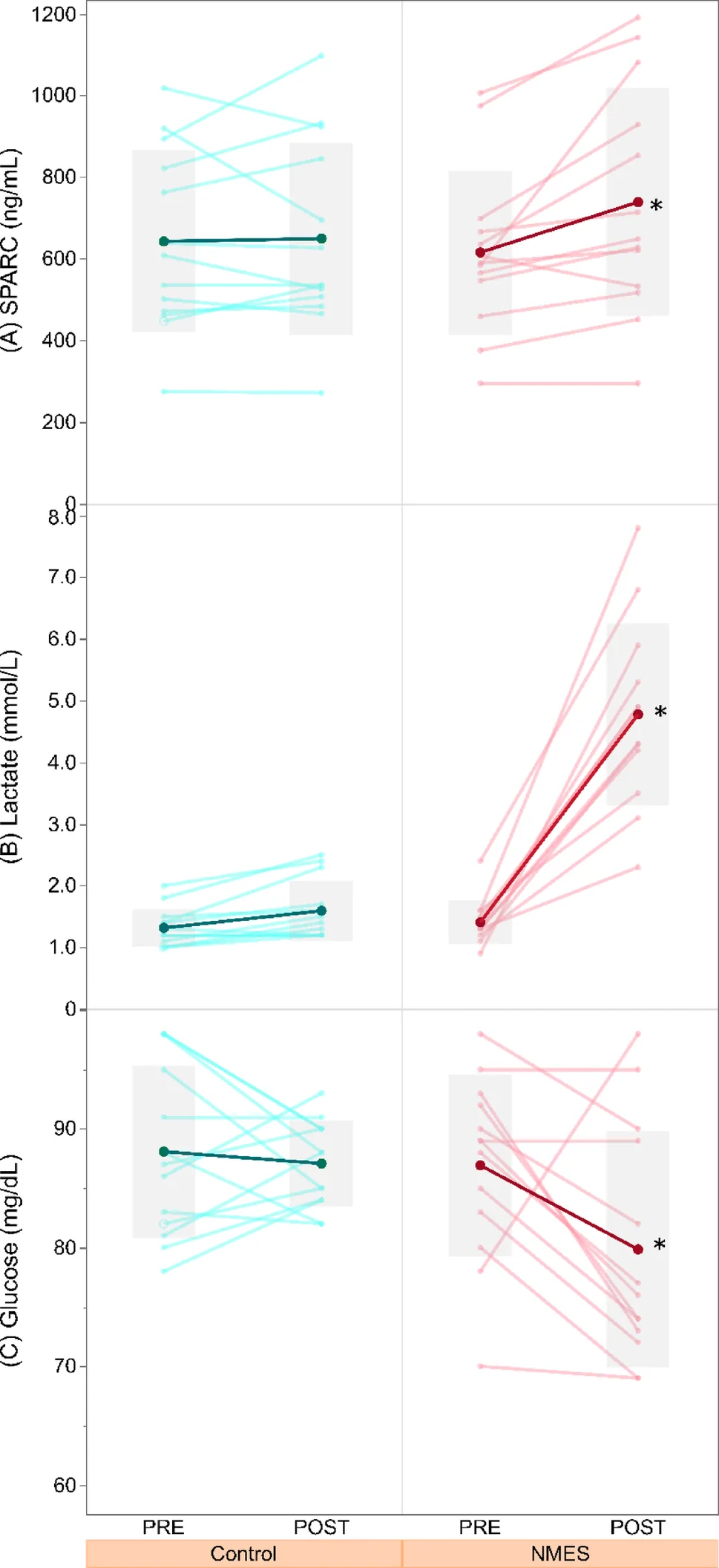
Acute responses of serum SPARC and metabolic parameters to Control and NMES sessions. **A** Serum secreted protein acidic and rich in cysteine (SPARC) concentration, **B** blood lactate concentration, and **C** blood glucose concentration before (PRE) and immediately after (POST) the 30-minute interventions. Bold lines with dark markers (teal for Control; dark red for NMES) represent group mean values, and shaded gray areas indicate the standard deviation. Thin light-colored lines (light blue for Control; pink for NMES) are for individual responses (*n* = 13). Significant session-by-time interactions were observed for SPARC and lactate. * *p* < 0.05 vs. PRE within the neuromuscular electrical stimulation (NMES) session (Tukey’s HSD post-hoc test)

As summarized in Table 2; Fig. 2, NMES induced significant alterations in SPARC and metabolic parameters, whereas no such changes were observed in Control session.

**Table 2:** Summary of SPARC and metabolic responses to control and NMES sessions

Variable	Session	Pre-intervention	Post-intervention	Interaction (F, *p*)	Effect size (d)
SPARC (ng/mL)	Control	642.9 ± 221.7	649.9 ± 234.8	*F* (1, 36) = 5.26, *p* = 0.0277	0.07
	NMES	616.0 ± 200.9	739.2 ± 279.8*		0.61
Lactate (mmol/L)	Control	1.3 ± 0.3	1.6 ± 0.5	*F* (1, 36) = 55.91, *p* < 0.0001	0.95
	NMES	1.4 ± 0.4	4.8 ± 1.5*		8.42
Glucose (mg/dL)	Control	88.1 ± 7.3	87.1 ± 3.6	*F* (1, 36) = 2.92, *p* = 0.0960	-0.15
	NMES	86.9 ± 7.7	79.8 ± 9.9*		-0.92

Data are presented as mean ± standard deviation (*n* = 13). Statistical significance was determined by using linear mixed model (Session, Time). Interaction (*p*) represents the *p*-value for the session-by-time interaction effect. Post-hoc comparisons (Tukey-HSD) showed significant increases in SPARC (*, *p* = 0.0078) and Lactate (*, *p* < 0.0001), and a significant decrease in Glucose (*, *p* = 0.0377) only in the NMES session

*NMES* neuromuscular electrical stimulation, *SPARC* secreted protein acidic and rich in cysteine

### Serum SPARC (Fig. 2A)

Serum SPARC concentrations significantly increased following the NMES session, rising from 616.0 ± 200.9 to 739.2 ± 279.8 ng/mL (MD = 123.2, 95% CI [26.7, 219.6], *p* = 0.0078, *d* = 0.61) whereas no significant changes occurred during the Control session (MD = 7.0, 95% CI [− 57.7, 71.6], *p* = 0.997, *d* = 0.07). Statistical analysis confirmed a significant session-by-time interaction (*F*(1, 36) = 5.26, *p* = 0.0277, *η*_*p*_^*2*^ = 0.128) and a main effect of the time (*F*(1, 36) = 6.61, *p* = 0.0144; *η*_*p*_^*2*^ = 0.155); however, no significant main effect of session was observed.

### Blood lactate (Fig. 2B)

A robust metabolic response was observed during NMES, characterized by a substantial accumulation of blood lactate: lactate levels increased from 1.4 ± 0.4 to 4.8 ± 1.5 mmol/L (MD = 3.4, 95% CI [2.6, 4.2], *p* < 0.0001, *d* = 8.42). In contrast, blood lactate remained at baseline levels throughout the Control session (1.3 ± 0.3 to 1.6 ± 0.5 mmol/L, MD = 0.3, 95% CI [0.1, 0.5], *p* = 0.7799, *d* = 0.95). These different responses were supported by a highly significant session-by-time interaction (*F*(1, 36) = 55.91, *p* < 0.0001; *η*_*p*_^*2*^ = 0.608) and significant main effects of both session (*F*(1, 36) = 62.78, *p* < 0.0001) and time (*F*(1, 36) = 77.73, *p* < 0.0001).

### Blood glucose (Fig. 2C)

Following the NMES intervention, glucose concentrations reduced from 86.9 ± 7.7 to 79.8 ± 9.9 mg/dL (MD = − 7.1, 95% CI [− 13.8, − 0.3], *p* = 0.0377, *d* = − 0.92). In the Control session, glucose levels showed no such reduction (MD = − 1.0, 95% CI [− 5.6, 7.9], *p* = 0.9784, *d* = − 0.15). Although the interaction between session and time did not reach statistical significance (*F*(1, 36) = 2.92, *p* = 0.0960, *η*_*p*_^*2*^ = 0.075), significant main effects were found for both session (*F*(1, 36) = 5.56, *p* = 0.0239) and time (*F*(1, 36) = 5.16, *p* = 0.0292).

### Correlation analysis

To explore the physiological drivers of SPARC secretion, we analyzed the relationship between the magnitude of SPARC elevation and markers of high metabolic demand during NMES. The correlation between the Δ Lactate and Δ SPARC during NMES did not reach significant (*r* = 0.37, *p* = 0.21). Similarly, no clear physiological coupling was observed between the Δ Glucose and Δ SPARC (*r* = 0.21, *p* = 0.50).

## Discussion

The main finding of this study was that NMES induced significant changes in circulating SPARC levels at an intensity corresponding to a HR at the VT_1_. This systemic demand (95.4 ± 13.3% of HR at VT_1_) was nearly identical to the intensity (96.0 ± 11.6% of HR at VT_1_) that failed to elicit a SPARC response during voluntary exercise in our previous study (Miyamoto et al. 2022). These results suggest that NMES can dissociate localized metabolic stress—characterized by accelerated glycolysis and lactate accumulation—from systemic cardiorespiratory load. This phenomenon fundamentally aligns with a decades-long consensus in the NMES field (Vints et al. 2025; Hoshiai et al. 2021; Truong et al. 2017; Hamada et al. 2003, 2004). While this unique metabolic-systemic dissociation has been widely utilized to optimize muscle strengthening and glycemic control, the present findings further suggest its potential relevance as a physiological foundation within the field of exercise oncology. This study is the first to demonstrate in human subjects that the metabolic bypass induced by NMES can effectively stimulate the secretion of SPARC, a distinct tumor-suppressive myokine.

The distinct pattern of SPARC secretion observed during NMES is likely attributable to the non-selective recruitment of both Type I and Type II muscle fibers (Maffiuletti et al. 2011; Jubeau et al. 2007). In voluntary exercise, recruiting high-threshold Type II fibers and accumulating lactate require high-intensity exertion. Although isolated maximal voluntary contractions can theoretically achieve the recruitment of high-threshold fibers at low systemic ${{\dot{\mathrm{V}}\mathrm{O}}}_{2}$ (Henneman et al. 1965), such high-intensity volitional exertion is often unfeasible for frail populations (Liu and Latham 2009; Stackhouse et al. 2001). However, NMES bypasses these volitional constraints to robustly activate Type II fibers, accelerating substantial glycogen utilization and lactate accumulation (Hamada et al. 2003; Sinacore et al. 1990). This unique characteristic enables NMES to deliver the necessary localized metabolic stress without requiring high-intensity systemic demand. In this study, although HR during NMES was that at VT_1_ levels, the lactate concentration after NMES reached 5.0 ± 1.3 mmol/L. Previous research indicates that blood lactate concentrations at VT_1_ during voluntary exercise are typically approximately 2.0 mmol/L (Prioux et al. 2000), and the workload at VT_2_ is characterized by a further increase in lactate levels, specifically 4.0 mmol/L (Pallares et al. 2016). Therefore, the lactate levels observed in this study (5.0 mmol/L) suggest that NMES-induced metabolic stress reached a level equivalent to or exceeding that typically observed at VT_2_ intensity, despite HR remaining at VT_1_. This result provides proof-of-concept that NMES effectively bypasses the systemic cardiorespiratory threshold usually required to achieve the metabolic conditions associated with SPARC release during voluntary exercise (Miyamoto et al. 2022).

NMES-induced metabolic stress appears to be the primary driver of SPARC release, likely mediated by the adenosine monophosphate-activated protein kinase (AMPK) signaling pathway (Aoi et al. 2019). As AMPK possesses a glycogen-binding domain, glycogen-depleting exercises are expected to markedly elevate blood SPARC levels via AMPK activation (Philp et al. 2012). Additionally, lactate has been implicated in molecular signaling pathways associated with AMPK activation (Cerda-Kohler et al. 2018). The significant increases in lactate and SPARC levels, coupled with decreased blood glucose levels observed in this study, support the hypothesis that NMES-induced glycolytic responses serve as key triggers for SPARC secretion. However, Songsorn et al. (2017) reported that a single 20-s supramaximal cycling sprint failed to increase SPARC release, suggesting that a brief exercise bout may be insufficient to trigger the necessary metabolic response. Therefore, a high cardiorespiratory demand, as reflected by measures such as ${{\dot{\mathrm{V}}\mathrm{O}}}_{2}$ and HR, does not appear to be the sole determinant of circulating SPARC levels; rather, these findings suggest that the accumulation of high local metabolic stress is a necessary trigger. Given that a significant correlation between changes in SPARC levels and lactate concentrations during voluntary exercise has been previously reported (Miyamoto et al. 2022), it is likely that the SPARC response during NMES was also triggered by factors related to accelerated glycolytic metabolism, such as glycogen depletion and subsequent lactate accumulation.

Our correlation analysis reinforces the idea that metabolic stress is the primary determinant of SPARC secretion, regardless of the contraction mode. Although the correlation between Δ Lactate and Δ SPARC during NMES did not reach statistical significance (*r* = 0.37, *p* = 0.21), this finding should be interpreted within the context of the study’s statistical power. Our *a* priori power analysis was specifically optimized to detect the primary session-by-time interaction effects (1- *β* = 0.80, *n* = 12), and thus the study may have been underpowered to confirm secondary correlation coefficients. However, the observed effect size (*r* = 0.37) is remarkably consistent with the *r* = 0.411 (*p* < 0.05) reported in our previous voluntary exercise study (Miyamoto et al. 2022). This near-identical magnitude and direction of these correlations strongly suggest that the underlying physiological coupling between accelerated glycolytic stress and SPARC secretion remains intact across different contraction modes, even though the current sample size precluded statistical significance for this secondary parameter. It is plausible that once a specific threshold of intramuscular glycolytic flux and subsequent lactate accumulation is achieved, skeletal muscle secretes SPARC via the same biological pathway between the two contraction modes. Furthermore, the lack of relationship between Δ Glucose and Δ SPARC (*r* = 0.21, *p* = 0.50), which aligns with our previous work, reinforces the interpretation that SPARC release is decoupled from systemic glucose homeostasis and instead reflects the local metabolic environment within the stimulated muscle.

This study excluded three participants (sub-threshold group) from the final analysis because NMES-induced pain/discomfort prevented them from reaching the predefined physiological threshold (80% of the HR at VT_1_). Addressing this intolerance remains a critical challenge for the future clinical application of NMES. Although it is generally suggested that higher levels of adipose tissue increase electrical impedance and sensory discomfort (Maffiuletti 2010; Petrofsky 2008), the sub-threshold group paradoxically exhibited a lower mean body fat percentage (14.0 ± 3.1%) compared to the completers (15.8 ± 6.1%). This finding suggests that total adipose tissue volume was not the primary determinant of NMES intolerance in this study. Instead, other factors, such as the regional distribution of fat relative to the electrodes (Gregory and Bickel 2005) or individual variations in pain sensitivity (Maffiuletti et al. 2018), may have played a more important role. Additionally, the lack of prior NMES experience among our participants likely amplified their perceived pain/discomfort, potentially limiting the achievable stimulation intensity and the subsequent metabolic response. Therefore, implementing structured habituation protocols may be essential for applying sufficient metabolic stress in this population. Progressive programming over multiple sessions may reduce sensory anxiety and increase the tolerance threshold (O’Connor et al. 2021), thereby allowing individuals to achieve the therapeutic intensities necessary to trigger SPARC secretion. Future research should prioritize the optimization of these familiarization protocols to enhance participant compliance and to further validate the SPARC response elicited by NMES.

From a clinical perspective, our results suggest that NMES could serve as a supplementary modality for eliciting intensity-dependent SPARC responses within the framework of tumor suppression, subject to further validation in populations where traditional exercise guidelines are difficult to achieve. Although current guidelines emphasize moderate-to-vigorous-intensity exercise (Clinton et al. 2020; Bull et al. 2020), such activity is often unrealistic for frail older adults. NMES may offer a physiological pathway to circumvent this barrier by inducing a localized metabolic environment comparable to that required for SPARC secretion, even when individuals remain in sedentary or supine positions. Although the anti-tumor potential of SPARC has been reported in mouse models (Aoi et al. 2013), it remains unclear whether the magnitude of elevation of SPARC levels observed in our healthy cohort is sufficient to elicit similar protective effects in humans and clinical settings. In addition, for patients undergoing chemotherapy who frequently experience severe fatigue, the theoretical possibility of receiving exercise-mimetic SPARC responses at the bedside is clinically intriguing and warrants future investigation. Importantly, while exogenous SPARC administration represents a potential alternative, endogenous induction via NMES-mediated muscle contraction offers a more physiological approach. This method likely triggers a synergistic release of various exercise-induced factors (Severinsen and Pedersen 2020) and maintains a localized autocrine/paracrine signaling environment, which systemic delivery may fail to replicate.

Despite these promising clinical prospects, to date, no longitudinal or epidemiological studies have directly demonstrated that NMES intervention reduces colon cancer incidence or progression. Furthermore, available evidence regarding intervention-induced alterations in blood SPARC levels remains inconsistent across the literature (Bettariga et al. 2026; Kim et al. 2022; Banitalebi et al. 2019; Songsorn et al. 2017; Aoi et al. 2013), although epidemiological data suggest the necessity of a high-intensity threshold to lower resting concentrations (Nishida et al. 2022). While our findings demonstrate that NMES can successfully mimic this required high-intensity stimulus to trigger a transient spike into the circulation, these data capture only acute physiological responses. Given that cancer prevention is fundamentally multifactorial, encompassing diverse pathways (Wang et al. 2025; McTiernan et al. 2019; Aleksandrova et al. 2017), the clinical applicability of NMES suggested in this study should be interpreted as a preliminary physiological framework. Nevertheless, further longitudinal investigations are warranted to validate the long-term efficacy of chronic NMES interventions.

This study has some limitations. First, we measured only blood markers of glucose, lactate, and SPARC, and no muscle biopsies were performed. Consequently, the reported blood values reflect the net balance between uptake and release within the circulation. Although SPARC gene expression in skeletal muscle has been associated with circulating SPARC levels (Aoi et al. 2013), these findings should be interpreted with caution. Second, our study included only young, healthy men, which may limit the generalizability of the findings to females, older populations, or those with underlying pathologies. Indeed, metabolic responses to NMES have been shown to differ significantly between sexes (Miyamoto et al. 2015). Additionally, baseline SPARC expression is highly sensitive to the homeostatic environment and may vary substantially with age, obesity, or malignancy (Ghanemi et al. 2020; Akutsu et al. 2020; Banitalebi et al. 2019; Scime et al. 2010). Given that SPARC is a multi-functional protein involved in diverse processes—including inflammation and glucose homeostasis—baseline levels may be paradoxically altered in different metabolic or clinical states (Ghanemi et al. 2023). Consequently, further research is required to evaluate the generalizability of these acute NMES-induced SPARC responses across diverse clinical cohorts. Finally, HR-based intensity monitoring may be confounded by thermoregulatory strain and sweating, potentially inducing cardiovascular drift (Wingo et al. 2012). However, the substantial blood lactate elevation observed here confirms that NMES successfully induced potent intramuscular metabolic stress, effectively bypassing these systemic cardiovascular fluctuations.

## Conclusion

This study indicates that NMES at an intensity corresponding to HR at VT_1_ increases circulating SPARC levels in healthy young men, likely via accelerated glycolytic flux and subsequent lactate accumulation. By achieving a metabolic environment comparable to that observed during VT_2_-level voluntary exercise despite low systemic demand, NMES may effectively bypass the conventional cardiorespiratory threshold required for SPARC release. Ultimately, our findings offer a fundamental physiological framework for future research into NMES as a strategic exercise-mimetic intervention to induce increases in circulating SPARC levels for individuals where strenuous volitional exertion is contraindicated.

## Data Availability

All data generated or analyzed during this study are included in this published article.

## Abbreviations

AMPK: Adenosine monophosphate-activated protein kinase
BMI: Body mass index
CI: Confidence interval
CV: Coefficient of variation
ECG: Electrocardiogram
ELISA: Enzyme-linked immunoassay
HR: Heart rate
HSD: Honestly significant difference
MD: Mean difference
NMES: Neuromuscular electrical stimulation
PA: Physical activity
P_ET_CO_2_: End-tidal carbon dioxide partial pressure
P_ET_O_2_: End-tidal oxygen partial pressure
SPARC: Secreted protein acidic and rich in cysteine
${{\dot{\mathrm{V}}\mathrm{CO}}}_{2}$: Carbon dioxide production
$${{\dot{\mathrm{V}}\mathrm{E}}}$$: Ventilation
$${{\dot{\mathrm{V}}\mathrm{O}}}_{2}$$: Oxygen uptake
VT_1_: First ventilatory threshold
VT_2_: Second ventilatory threshold
